# Identification of novel follicular dendritic cell sarcoma markers, FDCSP and SRGN, by whole transcriptome sequencing

**DOI:** 10.18632/oncotarget.14864

**Published:** 2017-01-27

**Authors:** Luisa Lorenzi, Claudia Döring, Tobias Rausch, Vladimir Benes, Silvia Lonardi, Mattia Bugatti, Elias Campo, José Cabeçadas, Ingrid Simonitsch-Klupp, Anita Borges, Jay Mehta, Claudio Agostinelli, Stefano Aldo Pileri, Fabio Facchetti, Martin-Leo Hansmann, Sylvia Hartmann

**Affiliations:** ^1^ Pathology Unit, Department of Molecular and Translational Medicine, University of Brescia, Brescia, Italy; ^2^ Dr. Senckenberg Institute of Pathology, Goethe University, Frankfurt am Main, Germany; ^3^ Genecore, European Molecular Biology Laboratory (EMBL), Heidelberg, Germany; ^4^ Haematopathology Section, Hospital Clinic, IDIBAPS, University of Barcelona, Barcelona, Spain; ^5^ Department of Pathology, Portuguese Institute of Oncology, Lisbon, Portugal; ^6^ Institute of Pathology, Medical University of Vienna, Vienna, Austria; ^7^ Histopathology, SRL Diagnostics, Mumbai, India; ^8^ Department of Experimental, Diagnostic and Specialty Medicine, Haematopathology Section, S. Orsola-Malpighi Hospital, University of Bologna, Bologna, Italy; ^9^ Unit of Diagnostic Haematopathology, European Institute of Oncology, Milan, Italy

**Keywords:** follicular dendritic cell sarcoma, whole transcriptome sequencing, immunohistochemistry, follicular dendritic cell-secreted protein, serglycin

## Abstract

Follicular dendritic cell (FDC)-sarcoma is a rare neoplasm with morphologic and phenotypic features of FDCs. It shows an extremely heterogeneous morphology, therefore, its diagnosis relys on the phenotype of tumor cells. Aim of the present study was the identification of new specific markers for FDC-sarcoma by whole transcriptome sequencing (WTS). Candidate markers were selected based on gene expression level and biological function. Immunohistochemistry was performed on reactive tonsils, on 22 cases of FDC-sarcomas and 214 control cases including 114 carcinomas, 87 soft tissue tumors, 5 melanomas, 5 thymomas and 3 interdigitating dendritic cell sarcomas. FDC secreted protein (FDCSP) and Serglycin (SRGN) proved to be specific markers of FDC and related tumor. They showed better specificity and sensitivity values than some well known markers used in FDC sarcoma diagnosis (specificity: 98.6%, and 100%, respectively; sensitivity: 72.73% and 68.18%, respectively). In our cohorts CXCL13, CD21, CD35, FDCSP and SRGN were the best markers for FDC-sarcoma diagnosis and could discriminate 21/22 FDC sarcomas from other mesenchymal tumors by linear discriminant analysis. In summary, by WTS we identified two novel FDC markers and by the analysis of a wide cohort of cases and controls we propose an efficient marker panel for the diagnosis of this rare and enigmatic tumor.

## INTRODUCTION

Follicular dendritic cell sarcoma (FDC-S) is the neoplastic proliferation of follicular dendritic cells (FDC) [1–2]. FDC belong to the stromal compartment of secondary lymphoid organs [3] and share crucial interaction capacities with the haematopoietic counterpart; both quality and effectiveness of immune responses depend from their integrity [4–6]. Therefore, FDC-S is included in the WHO classification of Haematopoietic tumors [1] and not in the WHO classification of Tumors of Soft Tissue and Bone [7] despite of its mesenchymal origin. The complex mechanisms mediating these functions are still largely unknown but new insights have been recently achieved in experimental models [4, 8–10].

FDC-S occurs in adults (median age 50 years), with no gender predilection, in both nodal and extranodal sites [11–15]. Its behavior is unpredictable; in a recent large review study, local recurrence and distant metastasis were reported to be equal to 28.1% and 27.2%, respectively [12].

On histology, FDC-S is characterized by extreme variability in cell composition and growth patterns, diagnosis is supported by the use of a panel of FDC-associated immunohistochemical markers combined with others, specific for epithelial neoplasms, soft tissue tumors and melanoma, to be used for exclusion [16]. Some differential diagnoses can be extremely challenging, particularly those entities typically prone to antigen loss (i.e. melanomas and undifferentiated carcinomas) the markers of which may be occasionally expressed by FDC-S (i.e. S100 and EMA) [16].

At present, the most widely used FDC-S markers are complement component receptor (CR) 1 (CD35), CR2 (CD21), Fc fragment of IgE receptor 2 (FCER2, CD23) and Clusterin. Recently, other proteins were identified including CXCL13 [17], D2-40 [18] and Claudin 4 [19]. Noteworthy, FDC-S frequently express a defective phenotype which may lead to misdiagnosis [12].

The molecular pathogenesis of FDC-S is largely unknown. Epidermal growth factor receptor signaling is known to support FDC-S proliferation [20–21]; mutations affecting the nuclear factor κ B pathway and cell cycle regulation were recently identified, by targeted next generation sequencing, in a subgroup of FDC-S [22]. Data on whole genome and whole transcriptome sequencing on this tumor are still lacking.

In order to explore the molecular and proteomic landscape of FDC-S we performed the first whole transcriptome sequencing analysis on two FDC-S cases. Pathway enrichment analysis was performed and new candidate immunohistochemical markers were identified and validated in a large cohort of FDC-S and controls.

## RESULTS

### Pathological features of FDC-S undergoing whole transcriptome sequencing

**Figure 1:**
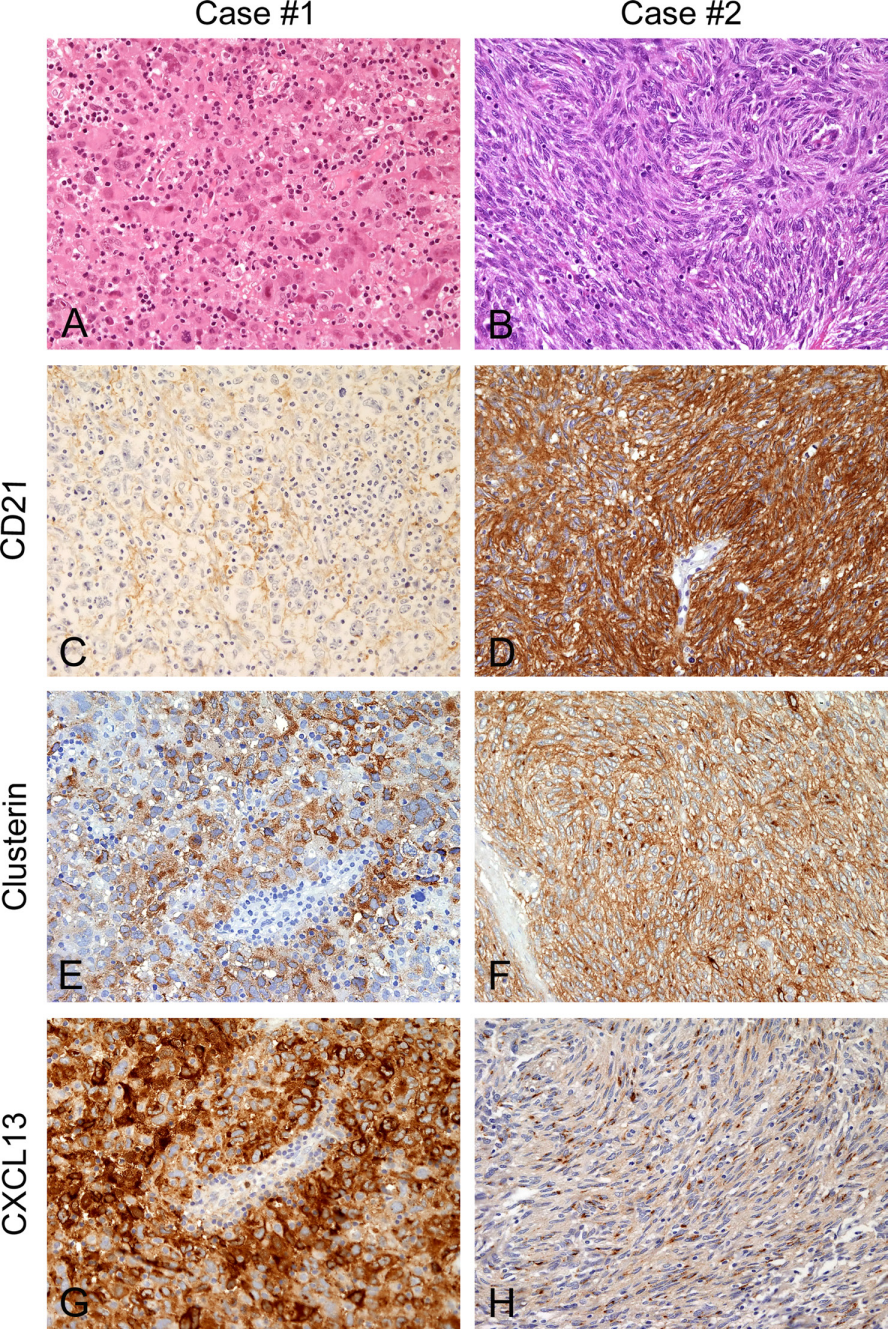
Morphology and phenotype of FDC-S cases submitted for transcriptome sequencing In Case #1 (**A**, **C**, **E** and **G**) atypical cells were epithelioid, pleomorphic, intermingled by numerous, small lymphocytes (A, hematoxylin and eosin), they stained negative for CD21 (C) but intensely expressed Clusterin (E) and CXCL13 (G). Case #2 (**B**, **D**, **F** and **H**) showed a different morphology with spindle atypical cells, arranged in fascicles (B, hematoxylin and eosin), positive for CD21 (D), Clusterin (F) and CXCL13 (H), the latter with a punctuate Golgi dot pattern. Magnification: 200×.

The clinical, morphological and phenotypical data of the two cases of FDC-S used for WTS are detailed in Table 1 and shown in Figure 1.

**Table 1:** Clinical, phenotypical and molecular data of FDC-S submitted for whole transcriptome sequencing

	Case #1		Case #2	
**Gender**	F		M	
**Age (years)**	71		76	
**Site**	Mediastinum		Soft tissues of the thigh	
**Maximum diameter**	5.9 cm		NA	
**Therapy and follow up**	Surgery and radiotherapy NED (15 months)		NA	
**FDC-S markers GENE_Protein**	protein expression by IHC	gene transcript (RPKM)	protein expression by IHC	gene transcript (RPKM)
CR2_CD21	–	49.0621	+	940.95374
FCER2_CD23	+	3519.927	+	295.29196
CR1_CD35	–	15.26415	–	54.7856
CLU_Clusterin	+	10482.2246	+	7388.229
PDPN_Podoplanin	+	213.73975	+	103.46978
CXCL13_CXCL13	+	8698.03906	+	1487.44849

Expression values of the genes encoding for CD21, CD23, CXCL13, CD35, Clusterin and Podoplanin correlate with immunohistochemical expression of their protein.

F, female; FDC-S, follicular dendritic cell sarcoma; IHC, immunohistochemistry; M, male; NA, not available; NED, no evidence of disease; RPKM, kilobase per million mapped reads.

The mediastinal biopsy of Case #1 showed highly pleomorphic cells, often multinucleated; with nuclear pseudo-inclusions and atypical mitoses. Reactive T and, less numerous, B- lymphocytes were intermingled within the tumor. Differently, in Case #2, tumor cells were bland looking, spindle, arranged in parallel fascicles; mitoses were few and never atypical, scattered mature lymphocytes were present. Tumor cell content was estimated on histological section to be above 40% in both cases. Their phenotype (reported in Table 1) showed expression of at least two FDC-specific markers.

### Whole transcriptome of FDC-S

No fusion transcripts were detected by whole transcriptome sequencing excluding the presence of chromosome translocations with oncogenic significance.

Genes with reads per kilobase per million mapped reads (RPKM) higher than 1 were 17,956 in case #1 and 18,118 in case #2. *IGK* (RPKM = 32,171.7754) and *FDCSP* (RPKM = 11,593.3535) had the highest expression value, in case #1 and #2, respectively.

Gene ontology and functional annotation of both lists generated an equivalent output, despite the differences in tissue type and tumor morphology.

Annotation of gene list for biological processes suggested a metabolically active tumor given enrichment of “metabolic process” (GO:0008152) and “cellular process” (GO: 0009987). Among the cellular components actin-related cytoskeleton (GO: 0015629) resulted enriched, in line with the actin-dependent activity of FDC [23]. Overrepresentation analysis was performed with the Panther pathway annotation dataset separately, in the two cases. Among others, a statistically significant (*p* < 0.05) overrepresentation of “Ubiquitin Proteasome” (P00060) pathway was detected in both cases, in line with the recent evidence of NFκB pathway regulatory-factors loss of function in this tumor [22]. According to the histological evidence of a higher T-cell infiltrate, “T-cell activation” (P00053) was increased in the transcriptome of case #1 while “Integrin Signalling” (P00034) and “Epidermal growth factor receptor (EGFR) signalling” pathway (P00018) were overrepresented in case #2, consistent with previous observations of activated EGFR signalling in FDC-S. [20–21]. Fold changes and *p*-values are listed in Supplemental material, Supplementary Table 1.

### Transcripts coding for established immunohistochemical FDC-S markers are among the top 10% of FDC-S transcriptome

Genes coding for FDC markers showed expression values higher than 55 RPKM and were among the “top 10%” genes of each transcriptome. Notably, *CLU* and *CXCL13* were those with the highest RPKM; *CLU* had the second transcription value in both; *CXCL13* was respectively at the 7^th^ and 36^th^ position (Supplementary Table 2). Accordingly, the transcriptome of the CD21-negative case #1 showed low expression values of the CD21-coding gene *CR2* and *CR1*, gene coding for CD35, had low RPKM values in both transcriptomes, in line with the negativity of both cases for this protein by immunohistochemistry (Table 1, Figure 1).

### *FDCSP* and *SRGN* have high expression values in FDC-S and are expressed by normal FDC

To identify additional markers for FDC-S we selected gene products with biological functions related to the germinal center reaction among the top 10% transcripts of both tumors.

**Figure 2:**
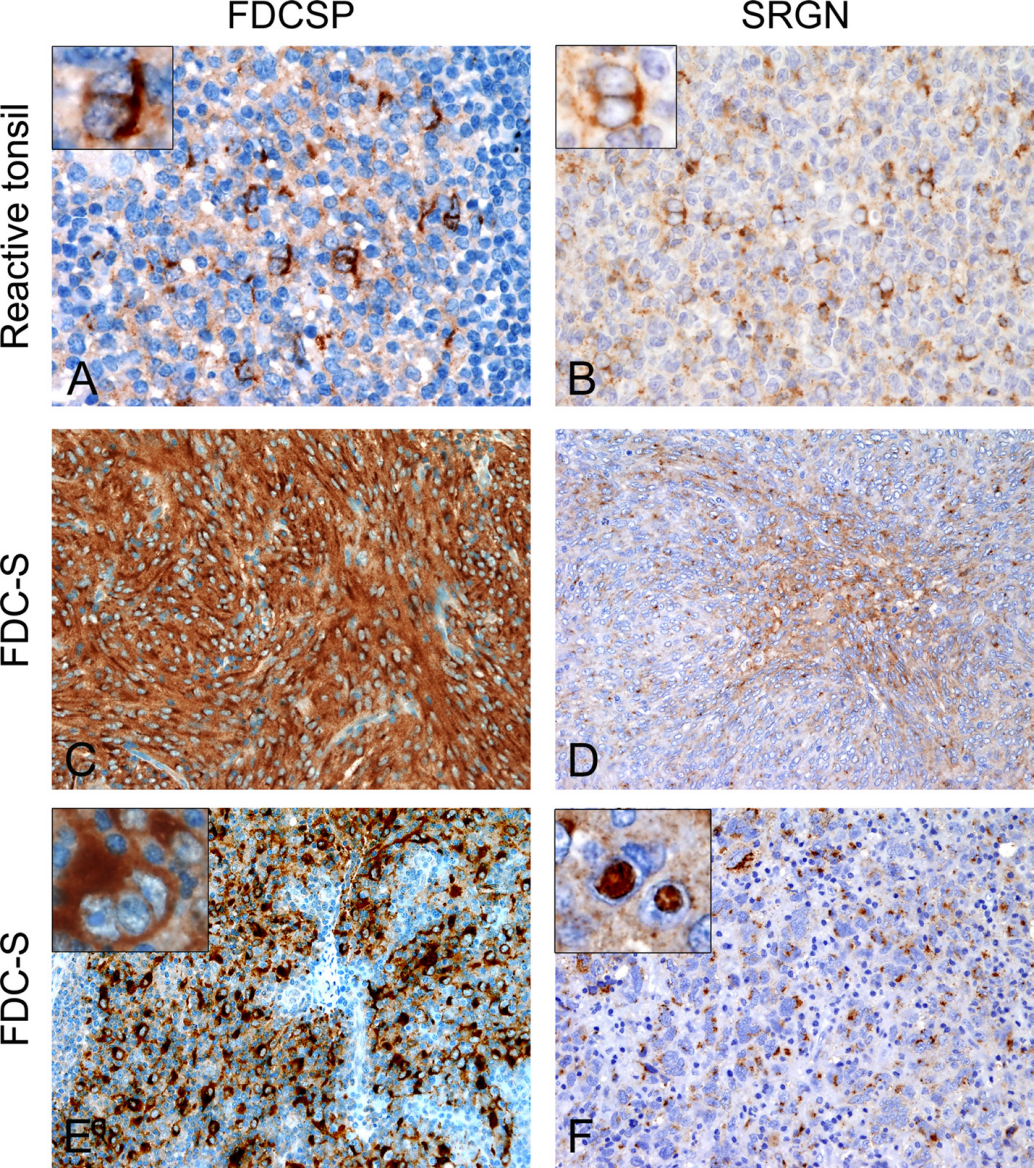
Expression patterns of FDCSP and SRGN on normal and neoplastic FDC In the contest of reactive germinal centers normal FDC react for both FDCSP and SRGN protein with a perinuclear expression pattern (**A**, **B**). FDCSP (**C**, **E**) and SRGN (**D**, **F**) are axpressed also in the cytoplasm of neoplastic FDC either with diffuse and intense staining or with a more delicate punctuate, cytoplasmic dot. SRGN often stained nuclear pseudo-inclusions (F, inset). Magnification: (A and B) 400×. (C, D, E and G) 200×. Insets: 600×. Abbreviations: FDC-S, follicular dendritic cell sarcoma; FDCSP, FDC secreted protein; SRGN, Serglycin.

Follicular dendritic cell secreted protein (*FDCSP*), also known as Chromosome 4 open reading frame 7*(C4Orf7)* and Serglycin (*SRGN*) were selected and antibodies against their proteins were tested on reactive tonsils. The cytoplasm of normal FDC, mostly located in the light zone of germinal centers of secondary lymphoid follicles, stained positive for both proteins with a perinuclear pattern (Figure 2A and 2B).

### FDCSP and SRGN exhibit good sensitivity and specificity for FDC-S diagnosis

Immunohistochemistry on follicular dendritic cell sarcomas for FDCSP was positive in sixteen cases (16/22, 72.73%) with either an intense and diffuse cytoplasmic staining or a prevailing perinuclear positivity. Reaction for SRGN was detected in fifteen cases (15/22, 68.18%) and, similarly to CXCL13, displayed a strong Golgi staining pattern with or without diffuse cytoplasmic reaction; notably, nuclear pseudo inclusions were frequently SRGN-positive (Figure 2).

In order to test the specificity of FDCSP and SRGN in FDC-S diagnosis we stained 87 soft tissue tumors, 5 thymomas, 5 melanomas (Supplementary Table 3), 3 interdigitating dendritic cell sarcomas and 114 carcinomas [24]. FDCSP was expressed by two soft tissue tumors, one sinonasal haemangiopericytoma (SNHP-1) and one synovial sarcoma (SS-2); SRGN was negative in all (Supplementary Table 4). Notably, one case of interdigitating dendritic cell sarcoma expressed FDCSP in about 40% of neoplastic cells while it was negative for SRGN and other FDC markers. No reactivity for FDCSP and SRGN was detected on melanomas, thymomas or carcinomas.

### CXCL13, CD21, CD35, FDCSP and SRGN are the best marker combination in FDC-S diagnosis

In order to compared the performance of FDCSP and SRGN with known FDC-S markers in the differential with soft tissue tumors, we tested CD21, CD23, CD35, CXCL13, Clusterin, Claudin 4 and Podoplanin in our groups of tumors and controls.

CXCL13, CD21 and Clusterin were the most retained markers in FDC-S with sensitivities of 90.91% for the first and 81.82% for the latter two (Table 2). CD35 and FDCSP were both expressed by 72.73% (16/22) of cases. The least sensitive were CD23 and Podoplanin, positive in 63.64% (14/22) of cases and Claudin 4, retained only by half of FDC-S (10/20). The highest specificity was observed for SRGN, CXCL13 and for complement and Fc receptors (CD21, CD35 and CD23): they were negative in all soft tissue tumors used as controls. Adversely, Clusterin showed a diffuse, undoubtful, expression in six cases of GIST (6/20, 30% not shown), all from extra-gastric sites. Podoplanin was expressed by two thirds of IMT cases (4/6, 66.67%, not shown), 25% (3/12) of SS, 15% (3/20) of GIST and 10.53% of SFT (2/19). Claudin 4 was expressed in one case of SS and one case of ALK-negative IMT.

**Table 2:** Sensitivity, specificity, area under the curve (AUC) and significance *p*-value of FDC markers

Marker	Sensitivity (+/FDC-S)	Specificity (–/STT)	AUC	*p*-value
**CXCL13**	90.91% (20/22)	100.00% (72/72)	0.955	**< 0.0001**
**CD21**	81.82% (18/22)	100.00% (72/72)	0.909	**< 0.0001**
**CD35**	72.73% (16/22)	100.00% (72/72)	0.864	**< 0.0001**
**FDCSP**	72.73% (16/22)	97.20% (70/72)	0.850	**< 0.0001**
**SRGN**	68.18% (15/22)	100.00% (72/72)	0.841	**< 0.0001**
**Clusterin**	81.82% (18/22)	81.94% (59/72)	0.810	**< 0.0001**
**CD23**	63.64% (14/22)	100.00% (72/72)	0.800	**< 0.0001**
**Podoplanin**	63.64% (14/22)	83.33% (60/72)	0.767	**< 0.0001**
**Claudin4**	50.00% (10/20)	98.61% (71/72)	0.736	**< 0.0001**

**Figure 3:**
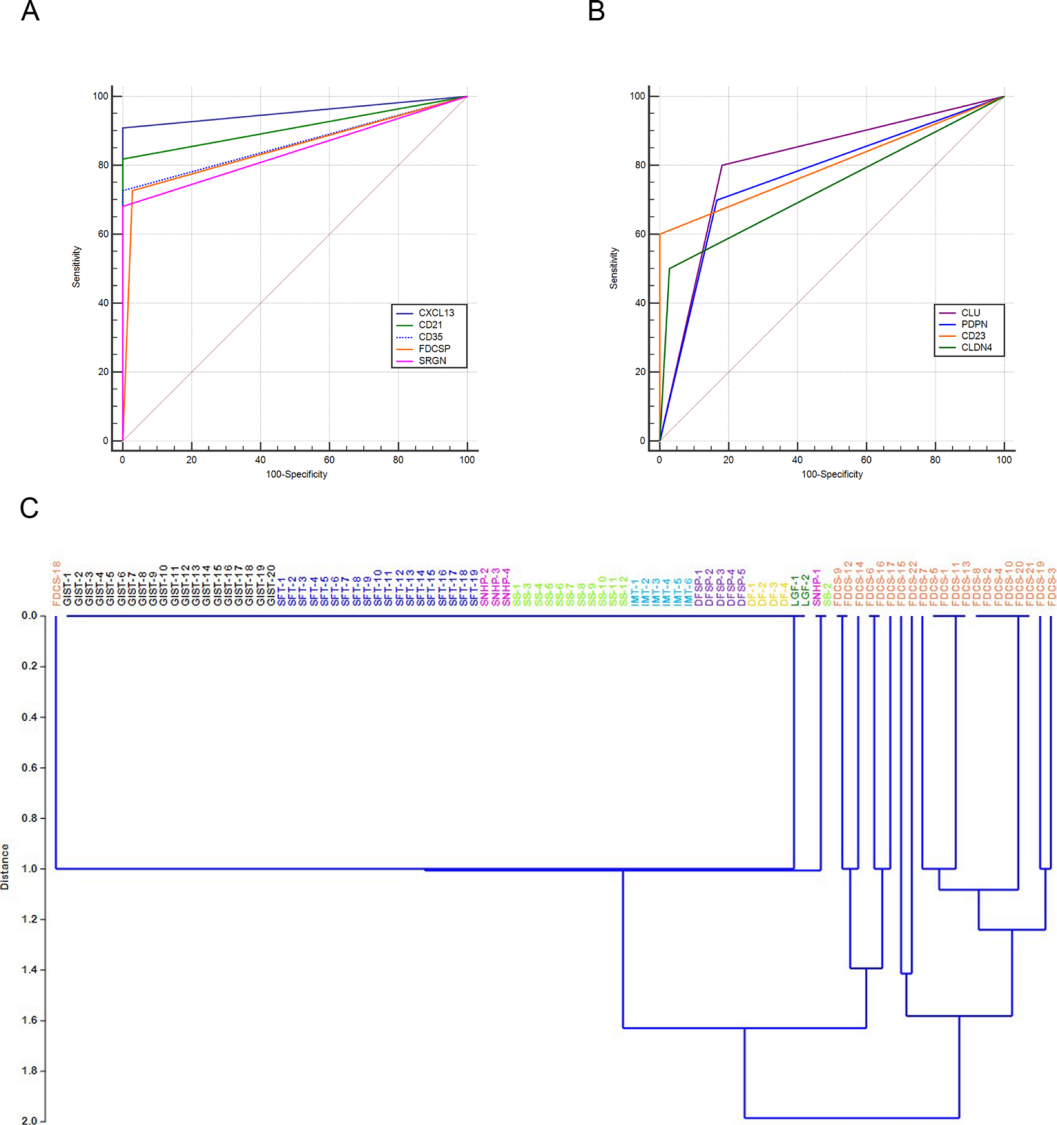
Representation of sensitivity and specificity of FDC markers by ROC curves and hierarchical clustering The graphs represent the AUC of FDC markers: those with best sensitivity and specificity values, CXCL13, CD21, CD23, FDCSP and SRGN are plotted in (**A**) Clusterin, Podoplanin, CD23 and Claudin 4 are displayed in (**B**). Hierarchical clustering performed considering IHC expression scores of the five markers CXCL13, CD21, CD23, FDCSP and SRGN in 22 FDC-S and 72 soft tissue tumors show a neat separation of FDC-S from controls with the exception of a single FDC-S case (**C**). Abbreviations: DF, desmoid type fibromatosis; DFSP, dermatofibrosarcoma protuberans; FDC-S, follicular dendritic cell sarcoma; GIST gastrointestinal stromal tumor; IMT, inflammatory myofibroblastic tumor; LGF, low grade fibromyxoid sarcoma; SFT, solitary fibrous tumor; SNHP, sinonasal haemangiopericytoma; SS, synovial sarcoma.

Sensitivity and specificity of each marker were plotted on a ROC curve and the value of their area under the curve (AUC) was calculated (Figure 3, Table 2).

Notably, CXCL13 showed the highest AUC, representing the marker with the best performance in the differential diagnosis of FDC-S with other tumors of mesenchymal origin. It was followed, in order, by CD21, CD35, FDCSP and SRGN (Figure 3A). Other widely used FDC-S markers such as Clusterin, CD23 and Podoplanin showed a lower AUC value (Figure 3B).

Evaluating the power of the combination of CXCL13, CD21, CD35, FDCSP and SRGN, by linear discriminant analysis (LDA), this marker combination showed a correct classification for 98.94% (93/94) of the cases tested, representing the best marker combination for the identification of FDC-S. When considering the same set of markers, FDC-S cases were nicely discriminated from the controls by principal component analysis (Supplementary Figure 1) with the exception of one case, FDC-S-18, by LDA (Figure 3C). The latter case developed as a retroperitoneal mass in a 73 years-old woman; neoplastic cells stained for Clusterin, Podoplanin and SRGN but were negative for all the other markers and may therefore represent an exceptional case.

## DISCUSSION

Follicular dendritic cell sarcoma (FDC-S) is a mesenchymal tumor with morphological and phenotypical features of follicular dendritic cells [1, 4, 12, 16]. FDC-S have been reported in numerous sites of the body, both nodal and extranodal [12]. The morphological appearance of FDC-S includes different growth patterns: fascicular, storiform, whorled or diffuse; puzzle-like, nodular (i.e. folliculocentric) and angiomatoid architectures have also been described [1, 25]. Neoplastic FDC are spindle, oval or epithelioid and often show the same characteristics of normal FDC but nuclear pleomorphism may also occur [1]. Given its heterogeneous morphology, FDC-S diagnosis is supported by the use of immunohistochemistry, it demands the usage of a wide panel of antibodies, given its frequent loss of specific antigens and the possibility of focal reactivity for epithelial markers [16–17]. FDC-S in both nodal and extranodal sites may mimic a wide spectrum of neoplasms including metastatic melanomas, poorly differentiated carcinomas, both epithelioid and spindle cell sarcomas. The use of recently identified antibodies, such as STAT6 for solitary fibrous tumor and TLE1 for synovial sarcoma, are helpful in some settings. However, not all entities have highly specific and retained markers and a panel of antibodies must be applied, still.

We performed whole transcriptome sequencing (WTS) on two cases of FDC-S in order to explore the biology of this rare tumor in terms of gene fusions and gene expression.

Our study did not identify fusion transcripts in the two cases of FDC-S analyzed excluding the presence of chromosome translocations of oncogenic significance in these patients. However, we could use expression values obtained from WTS to identify two novel immunohistochemical markers for both normal and neoplastic FDC: Follicular dendritic cell secreted protein (FDCSP) and Serglycin (SRGN).

FDCSP, isolated in 2002 from human reactive tonsils, is specifically expressed by B-cell-activating-FDC [26]. The few studies performed to understand the functions of this protein suggest that it can modulate B-cell immune response [26–27] and may have a role in autoimmune diseases [28–29]. SRGN is an intracellular proteoglycan implicated in secretory granules formation and immune response modulation as demonstrated in knock-out mouse models [30–31]. *SRGN* expression was reported in the transcriptome of different cell types, including stromal and endothelial cells [30]. Notably, both markers were previously associated with metastasis and dismal prognosis in a wide range of carcinomas [31–36] but we could not detect their expression, by immunohistochemistry, in our cohort of controls.

In FDC-S diagnosis, FDCSP and SRGN showed both a good sensitivity, higher than CD23 and Claudin 4, and an extremely good specificity, evaluated on 214 controls including carcinomas, soft tissue tumors, melanomas, thymomas and interdigitating dendritic cell sarcomas. SRGN, together with CD21 and CD35, was negative in all non-FDC-S tumors; FDCSP, expressed by one case of SNHP, one case of SS and one case of interdigitating dendritic cell sarcoma, still showed a better performance than other widely used FDC markers. Clusterin, highly expressed at transcriptome level, is a highly sensitive marker for FDC-S [37–38]. However, in our study, it stained also 60% of GISTs occurring in the GI tract, outside stomach (e.g. ileum wall), suggesting to use it with caution (eventually together with CD117 and DOG1). Meanwhile, Podoplanin, a sensitive marker for FDC-S [18], was expressed in two thirds of IMT, in a fraction of GIST, SFT and SS. Pathologists should be aware of these potential pitfalls in the differential diagnosis of FDC-S occurring in extranodal sites and should consider applying also other newly, highly specific markers, such as STAT6 [39] and TLE1 [40], in support.

In our study the combination of CXCL13, CD21, CD35, FDCSP and SRGN reached a very good discriminatory power in distinguishing FDC-S from other soft tissue tumors. In particular, CXCL13, a chemokine ligand recently reported in FDC-S [17], was the marker with best sensitivity and specificity combination (Table 2). The usefulness of our five marker combination was confirmed by the good results obtained by linear discriminant analysis and the almost perfect separation in the hierarchical clustering profile where all FDC-S cases, but one, grouped together. Notably, all the correctly classified FDC-S cases were positive for at least two markers among CXCL13, CD21, CD35, FDCSP and SRGN; this information could be easily applicable in the routine diagnosis of this tumor.

FDC-S with a highly defective phenotype do exist and were present also in our set of cases, this could be related to a different grade of differentiation or “activation” of neoplastic FDCs and suggests to further explore the biology of these tumors in correlation with other mesenchymal tumors with the same, putative, cell of origin [9].

In conclusion, by a combined approach of next generation sequencing and high throughput immunohistochemistry we identified two novel markers for FDC-S, FDCSP and SRGN, and suggested a highly effective and ready-to-use approach for its differential diagnosis with other mesenchymal tumors.

## MATERIALS AND METHODS

### Case selection

Twenty-two cases of follicular dendritic cell sarcoma cases (FDC-S) were retrieved from the archives of pathology of the University Hospitals of Brescia, Frankfurt am Main, Bologna, Lisbon, Barcelona, Vienna and Mumbay. Diagnosis were confirmed at a multi head microscope by the authors (C.A., F.F, M.L.H., S.H. and S.A.P.).

Eighty-seven cases of soft tissue tumors were retrieved from the archives of the department of pathology of Spedali Civili di Brescia. They included 19 Solitary fibrous tumors (SFT, all STAT6-positive) and 4 Sinonasal haemangiopericytoma (SNHP, STAT6- negative [41]), 20 gastrointestinal stromal tumors (GIST, DOG1 and/or CD117-positive), 12 synovial sarcomas (SS, all TLE1-positive, 9 with split of 18q11.2, evaluated by fluorescence *in situ* hybridization with SS18 probe), 6 inflammatory myofibroblastic tumors (IMT, 4 ALK-positive), 5 dermatofibrosarcomas protuberans (DFSP), 5 leiomyosarcomas (LMS), 5 angiosarcomas (AS), 4 desmoid-type fibromatosis (DTF), 3 undifferentiated pleomorphic sarcomas (UPS), 2 dedifferentiated liposarcomas (DLS) and 2 low-grade fibromyxoid sarcomas (LGFMS, all MUC4-positive). Cases were revised and diagnosis confirmed by expert soft tissue pathologists (Demographical data are detailed in Supplementary Table 3). Informed consent was obtained according to the Declaration of Helsinki and the local ethics committees agreed on the study.

### RNA extraction from frozen material and whole transcriptome sequencing

Total ribonucleic acid (RNA) was extracted from the frozen tissue of four FDC-S (RNeasy Mini kit, Qiagen, Hilden, Germany). RNA integrity was evaluated by a Bioanalyzer (Agilent, Waldbronn, Germany) and two of the four cases showed sufficient quality for whole transcriptome sequencing (WTS). The latter was performed on an Illumina HiSeq platform (EMBL, Heidelberg, Germany) after library preparation with the SMARTer™ Ultra Low RNA Kit (Clontech, Mountain View, CA USA). The hg19 Genome Reference Consortium GRCh37 was used as reference genome applying the Genomatix Software (Genomatix GmbH. Munich, Germany) using default parameters.

### Biostatistical analysis

Transcriptome data were analyzed for fusion transcripts by Genomatix Mining Station and Genome Analyzer.

Gene expression values were normalized and ordered per kilobase per million mapped reads (RPKM). Genes with RPKM higher or equal to 1 underwent gene annotation analysis by Gene Ontology Consortium-annotations-based Panther software (www.pantherdb.org) [42]; overrepresentation test (by bimodal test with Bonferroni correction) and enrichment test (by Mann-Whitney test) were performed on both Panther and Genomatix softwares.

After immunohistochemical analysis, sensitivity, specificity and corresponding *p*-values were calculated for candidate and known FDC-S markers. Receiver Operating Characteristic (ROC) curve analysis was performed with the ©1993–2016 MedCalc Software using the methodology detailed in DeLong *et al*. [43] for calculation of the Standard Error and the exact Binomial Confidence Interval for the Area Under the Curve. Hierarchical clustering, principal component analysis (PCA) and linear discriminant analysis were performed with the PAST software [44].

### Tissue microarray preparation and immunohistochemistry

Tissue microarray (TMA) blocks were created from FDC-S and 72 controls (control group #1, Supplementary Table 3), using an automated tissue microarray (TMA Master, 3DHistech, Budapest, Hungary). Three representative tumor cores (1 or 1.5 mm diameter) were identified on hematoxylin and eosin (H&E) stained sections and punched from the original tissue blocks.

Immunohistochemistry for known and novel FDC markers was performed on these TMAs. In order to further confirm their specificity, FDCSP and SRGN were additionally applied on TMAs including 114 carcinomas of different origin, previously described [24], and on whole tissue sections of 28 additional control cases (control group #2, Supplementary Table 3). Antibodies applied are detailed in Supplementary Table 5.

Immunohistochemistry for FDCSP and SRGN was performed with automatic immunostainer (Dako, Glostrup, Denmark) or manually, showing equivalent results; immunohistochemistry on controls was performed automatically (Leica Biosystems, Wetzlar, Germany).

Stained sections were digitalized using the Aperio Scanscope System (Leica Biosystems) and evaluated by two pathologists (S.H. and L.L.). Cases were scored positive when reaction occurred on at least 30% of tumor cells.

## SUPPLEMENTARY MATERIALS FIGURES AND TABLES


