# Supplementary material for: Identification of novel follicular dendritic cell sarcoma markers, FDCSP and SRGN, by whole transcriptome sequencing
Source: Oncotarget. 2017 Jan 27;8(10):16463–72. doi: 10.18632/oncotarget.14864 (PMC5369977; doi:10.18632/oncotarget.14864)
Supplement: Supplementary file 1 [file oncotarget-08-16463-s001.pdf]

# Identification of novel follicular dendritic cell sarcoma markers, FDCSP and SRGN, by whole transcriptome sequencing

## Supplementary Materials

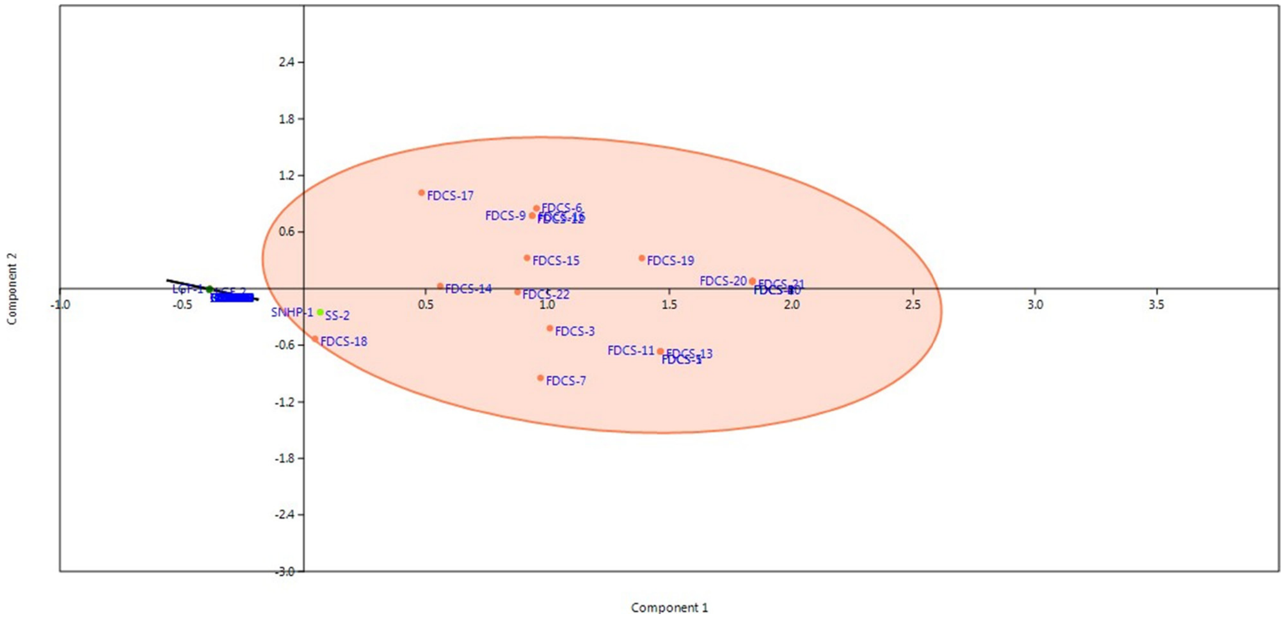


**Supplementary Figure 1: Principal component analysis performed considering the immunohistochemical evaluation of CD21, CD35, CXCL13, FDCSP and SRGN on 22 follicular dendritic cell sarcomas and 72 soft tissue tumors.** Eclipses indicate the area including 95% of cases of a given category (follicular dendritic cell sarcoma in orange). Abbreviations: FDCS, follicular dendritic cell sarcoma; LGF, low grade fibromyxoid sarcoma; SNHP, sinonasal haemangiopericytoma; SS, synovial sarcoma.

**Supplementary Table 1: Pathway enrichment analysis of genes with RPKM values higher than 1**

| | Homo Sapiens (reference) | Case #1 | | | | |
|---|---|---|---|---|---|---|
| **PANTHER pathways** | **# genes** | **# genes** | **# expected** | **FE** | **+/-** | ***p*-value** |
| Apoptosis signaling pathway (P00006) | 115 | 104 | 68.17 | 1.53 | + | **4.82E-03** |
| Ubiquitin proteasome pathway (P00060) | 63 | 63 | 37.35 | 1.69 | + | **1.20E-02** |
| Parkinson disease (P00049) | 107 | 96 | 63.43 | 1.51 | + | **1.24E-02** |
| T cell activation (P00053) | 80 | 74 | 47.43 | 1.56 | + | **3.19E-02** |
| | **Homo Sapiens (reference)** | **Case #2** | | | | |
| **PANTHER pathways** | **# genes** | **# genes** | **#expected** | **FE** | **+/-** | ***p*-value** |
| Apoptosis signaling pathway (P00006) | 115 | 106 | 69.99 | 1.51 | + | **5.38E-03** |
| Integrin signalling pathway (P00034) | 181 | 152 | 110.15 | 1.38 | + | **1.32E-02** |
| Ubiquitin proteasome pathway (P00060) | 63 | 62 | 38.34 | 1.62 | + | **4.06E-02** |
| EGF receptor signaling pathway (P00018) | 130 | 112 | 79.12 | 1.42 | + | **4.21E-02** |
| Parkinson disease (P00049) | 107 | 95 | 65.12 | 1.46 | + | **4.51E-02** |

FE, fold enrichment. Performed by Panther (www.pantherdb.com).

**Supplementary Table 2: Top 10% gene list from whole transcriptome sequencing of two cases of follicular dendritic cell sarcoma.** RPKM, reads per kilobase per million mapped reads. See Supplementary_Table_2

**Supplementary Table 3: Demographical data of follicular dendritic cell sarcomas (patients) and cases, used as controls (controls #1 and #2), divided by group of entity**

| Group | Diagnosis | Nr | Gender M:F | Median age in years (range) | Site |
|---|---|---|---|---|---|
| **patients** | **Follicular dendritic cell sarcoma** | **22** | 10:12 | 63 (35–84) | Lymph node (8), retroperitoneum (4), lung (2), soft tissue (2), spleen (2), epipharynx (1), colonic wall (1), liver (1), parotid gland (1) |
| **controls #1** | **Solitary fibrous tumour** | **19** | 7:12 | 49 (41–75) | pleura (7), SNC (2), soft tissues of the extremities (2), mesentery (1), retroperitoneum (2), larynx (1), lung (1), omentum (2), oral cavity (1), vulva (1) |
| **controls #1** | **Sinonasal haemangiopericytoma** | **4** | 2:2 | 58.5 (61–79) | Sinonasal cavity (4) |
| **controls #1** | **Gastrointestinal stromal tumor** | **20** | 5:15 | 64 (47–87) | Stomach (10), ileum (6), duodenum (1), esophagus (1), mesentery (1), rectum (1) |
| **controls #1** | **Synovial sarcoma** | **12** | 6:6 | 45.5 (9–64) | Soft tissues of the extremities (5), retroperitoneum (2), lung (1), mediastinum (1), mesentery (1), nasal fossa (1), pleura (1) |
| **controls #1** | **Inflammatory myofibroblastic tumor** | **6** | 5:1 | 40 (21–58) | Lung (2), ileum (1), mediastinum (1), omentum (1), trachea (1) |
| **controls #1** | **Dermatofibrosarcoma protuberans** | **5** | 4:1 | 51 (23–52) | Subcutis (5) |
| **controls #1** | **Desmoid-type fibromatosis** | **4** | 2:2 | 50 (13–69) | Subcutis (3), ileal wall (1) |
| **controls #1** | **Low grade fibromyxoid sarcoma** | **2** | 1:1 | 57 (42–72) | Subcutis (2) |
| **controls #2** | **Angiosarcoma** | **5** | 3:2 | 55 (40–70) | Breast (2), skin (2), lung (1) |
| **controls #2** | **Leiomyosarcoma** | **5** | 0:5 | 62 (49–75) | Subcutis (2), uterus (2), head and neck (1) |
| **controls #2** | **Melanoma** | **5** | 4:1 | 63 (37–79) | Skin (4), lung (1) |
| **controls #2** | **Thymoma** | **5** | 1:4 | 69 (51–84) | Mediastinum (5) |
| **controls #2** | **Undifferentiated pleomorphic sarcoma** | **3** | 2:1 | 72 (64–79) | Subcutis (2), parotid gland (1) |
| **controls #2** | **Interdigitating dendritic cell sarcoma** | **3** | 1:2 | 70 (50–79) | Lymph node (2), oral cavity (1) |
| **controls #2** | **Dedifferentiated liposarcoma** | **2** | 2:1 | 57 (46–68) | Abdominal wall (1), soft tissues of the arm (1) |

**Supplementary Table 4: Number of cases expressing Follicular dendritic cell secreting protein (FDC-SP), Serglycin (SRGN), CD21, CD23, CD35, CXCL13, Clusterin (Clu), Claudin 4 (Cl4), Podoplanin (D2-40), by immunohistochemistry, in each group of entity**

| Tumor type | No. | % positive cases (No. positive / No. total cases analyzed) | | | | | | | | |
|---|---|---|---|---|---|---|---|---|---|---|
| | | FDCSP | SRGN | CD21 | CD23 | CD35 | CXCL13 | Clu | Cl 4 | D2–40 |
| **Follicular dendritic cell sarcoma** | **22** | 72.73% (16/22) | 68.18% (15/22) | 81.82% (18/22) | 63.64% (14/22) | 72.73% (16/22) | 90.91% (20/22) | 81.82% (18/22) | 50% (10/20) | 63.64% (14/22) |
| **Synovial sarcoma** | **12** | 8.33% (1/12) | 0% (0/12) | 0% (0/12) | 0% (0/12) | 0% (0/12) | 0% (0/12) | 25% (3/12) | 8.33% (1/12) | 25% (3/12) |
| **Gastrointestinal stromal tumor** | **20** | 0% (0/20) | 0% (0/20) | 0% (0/20) | 0% (0/20) | 0% (0/20) | 0% (0/20) | 30% (6/20) | 0% (0/20) | 15% (3/20) |
| **Solitary fibrous tumor** | **19** | 0% (0/19) | 0% (0/19) | 0% (0/19) | 0% (0/19) | 0% (0/19) | 0% (0/19) | 5.26% (1/19) | 0% (0/19) | 10.53% (2/19) |
| **Sinonasal haemangio-pericytoma** | **4** | 25% (1/4) | 0% (0/4) | 0% (0/4) | 0% (0/4) | 0% (0/4) | 0% (0/4) | 50% (2/4) | 0% (0/4) | 0% (0/4) |
| **Inflammatory myofibroblastic tumor** | **6** | 0% (0/6) | 0% (0/6) | 0% (0/6) | 0% (0/6) | 0% (0/6) | 0% (0/6) | 0% (0/6) | 16.67% (1/6) | 66.67% (4/6) |
| **Dermatofibro-sarcoma protuberans** | **5** | 0% (0/5) | 0% (0/5) | 0% (0/5) | 0% (0/5) | 0% (0/5) | 0% (0/5) | 0% (0/5) | 0% (0/5) | 0% (0/5) |
| **Desmoid-type fibromatosis** | **4** | 0% (0/4) | 0% (0/4) | 0% (0/4) | 0% (0/4) | 0% (0/4) | 0% (0/4) | 0% (0/4) | 0% (0/4) | 0% (0/4) |
| **Low grade fibro-myxoid sarcoma** | **2** | 0% (0/2) | 0% (0/2) | 0% (0/2) | 0% (0/2) | 0% (0/2) | 0% (0/2) | 50% (1/2) | 0% (0/2) | 0% (0/2) |

**Supplementary Table 5: Antibodies and probes used in the study**

| Antigen | Species/Clone | Dilution | Company |
|---|---|---|---|
| **ALK** | Mouse/5A4 | 1:30 | TS |
| **CD21** | Mouse/1F8 | 1:30 | DB |
| **CD23** | Mouse/1B12 | 1:20 | TS |
| **CD35** | Mouse/Ber-MAC-DRC | 1:50 | DK |
| **CD117** | Rabbit polyclonal | 1:100 | DK |
| **Claudin 4** | Mouse/3E2C1 | 1:200 | IN |
| **Clusterin** | Mouse/7D1 | 1:200 | LM |
| **CXCL13 (BCA-1)** | Goat polyclonal | 1:50 | RD |
| **DOG1** | Rabbit/SP31 | 1:100 | TS |
| **FDCSP** | Rabbit polyclonal | 1:35 | OR |
| **MUC4** | Mouse/8G7 | 1:200 | BC |
| **Podoplanin** | Mouse/D2-40 | 1:40 | DK |
| **Serglycin (SRGN)** | Rabbit polyclonal | 1:100 | SA |
| **STAT6 (S.20)** | Rabbit polyclonal | 1:200 | SC |
| **TLE1** | Mouse/1F5 | 1:30 | CM |

| FISH Probe | Target | | Company |
|---|---|---|---|
| **Break apart SS18** | Vysis 18q11.2 | – | AL |

AL, Abbott Laboratories, Lake Bluff, IL, USA; BC: Biocare Medical; Concord, CA, USA; CM: Cell Marque, Rocklin, CA, USA; DB: Diagnostic Biosystems, Pleasanton, CA, USA; DK: Dako, Glostrup, Denmark; LM: Leica Microsystems, Wetzlar, Germany; TS: Thermo Scientific, Fremont, CA, USA; OR, Origene, Rockville, Maryland, USA; RD: R&D Systems, Minneapolis, CA, USA; IN: Invitrogen, Carlsbad, CA, USA; SA: Sigma Aldrich, S. Louis, MO, USA; SC: Santa Cruz Biotechonology, Dallas, TX, USA.
